# Characterizing the causes and consequences of calcium oxalate crystal presence in *Vitis riparia*

**DOI:** 10.1002/ajb2.70173

**Published:** 2026-03-09

**Authors:** Carolyn D. K. Graham, Samantha Molino, Addison L. Yerks, Marjorie Weber

**Affiliations:** ^1^ Department of Ecology and Evolutionary Biology University of Michigan Ann Arbor 48109 Michigan USA; ^2^ Department of Biological Sciences University at Buffalo Buffalo 14260 New York USA

**Keywords:** biomineralization, druse, herbivory bioassay, induced defense, raphide, Vitaceae

## Abstract

**Premise:**

Calcium oxalate biomineralization in plants is phylogenetically widespread and morphologically diverse, but the function of these inorganic crystals is an area of active debate. The variety of environmental conditions that produce the crystals, as well as the inconsistent evidence that they provide antiherbivore defense across plant and herbivore species, suggests that different crystal morphologies might have different functions.

**Methods:**

Using *Vitis riparia*, or riverbank grape, we experimentally investigated the environmental influence of excess calcium and simulated herbivory on the formation of calcium oxalate druse and raphide crystals in leaves. We also investigated the putative defensive function of these crystals by using a no‐choice herbivore bioassay manipulating herbivore diet composition to test for impacts of crystal shape on herbivore growth, both on its own and with plant chemistry.

**Results:**

We found that the addition of calcium to soil increased the density of both raphide and druse crystals in *V. riparia* leaves. Contrary to expectations, the herbivory treatment decreased the density of raphides in leaves, and *V. riparia*‐derived crystals did not impact weight gain, time to pupation, or survival of moth larvae.

**Conclusions:**

Our multifaceted test of the formation and function of calcium oxalate crystals in riverbank grape demonstrates that an abiotic factor (i.e., soil calcium) is a relatively stronger determinant of crystal production and that, contrary to hundreds of years of speculation on their function, these crystals do not seem to mediate plant‐insect herbivory in all plant taxa. Instead, the alternative hypothesis of calcium regulation was supported by our experimental evidence.

Since calcium oxalate (CaOx) crystals were first discovered via microscope by Antonie van Leeuwenhoek in the 1600 s, plant biologists have hypothesized about their formation and functions (van Leeuwenhoek, 1675; Webb, 1999; Franceschi and Nakata, 2005). Nearly 350 yr later, the ecological role of CaOx crystals is still an area of active debate (Paiva, 2019, 2021; Khan et al., 2023; Lawrie et al., 2023). Plants in ≥200 families have CaOx crystals within their leaves, stems, roots, flowers, and/or fruits (McNair, 1932; Franceschi and Nakata, 2005), taking on a multitude of forms, from sharp needles to prisms to conglomerations (Webb, 1999; Raman et al., 2014). Many hypotheses have been proposed for the potential selective pressures that produce and maintain this variation in their morphologies (e.g., Volk et al., 2002; Park et al., 2009; Konno et al., 2014), but we lack manipulative experiments that simultaneously test the causes and consequences of CaOx presence in plant organs.

Calcium oxalate crystal expression is often hypothesized and demonstrated to hinge on the presence of excess calcium in a plant's environment. Calcium is an essential nutrient in plants that plays a variety of structural and signaling roles (White and Broadley, 2003). It can be found in abundance as freely exchangeable cations in many soil types, and indeed in overabundance to the point of plant cytotoxicity in heavily calcareous soils (McLean, 1975; White and Broadley, 2003). Calcium oxalate crystals have thus been proposed to function as dynamic storage for excess calcium (Paiva, 2019), but evidence for increased calcium oxalate crystal production in high‐calcium soil conditions is inconsistent and may be species specific. For instance, soil calcium concentration did not impact calcium oxalate production in *Pancratium sickenbergeri*, indicating that the crystals are not inducible in this plant species (Ruiz et al., 2002). Paradoxically, Molano‐Flores (2001) showed that *Sida rhombifolia* seedlings that were subjected to calcium‐scarce conditions had the highest quantities of druse crystals in their plant parts. Thus, the degree to which soil calcium plays a role in crystal presence and density is difficult to generalize across taxa.

One of the most compelling additional hypotheses for the presence of calcium oxalate crystals in plants is that they act as a defense against chewing herbivores. Herbivory is a strong selective pressure for plants due to their inability to move to escape predation. As a result, plants have evolved a diversity of traits that deter potential antagonists. Calcium oxalate crystals are proposed to be a physical deterrent to herbivory, causing internal or external irritation or abrasion to potential herbivores (Nakata, 2003; Franceschi and Nakata, 2005; He et al., 2014). Indeed, the sharp, abrasive nature of these crystals has long been implicated in dental wear and external and internal membrane irritation in mammals, including humans (Perera et al., 1990; Gardner, 1994; Danielson and Reinhard, 1998; Salinas et al., 2001). In the case of arthropod herbivores (a major group of plant antagonists), the picture is less clear and the presence of traits that protect against internal damage may modify this prediction for certain feeding guilds of arthropods (see discussion of the peritrophic matrix below; Paiva, 2021). Some researchers have found clear fitness impacts on soft‐bodied, chewing lepidopteran larvae that are fed calcium oxalate crystals (e.g., Korth et al., 2006; Park et al., 2009; both using *Spodoptera exigua*, a generalist herbivore), while others have not (e.g., Nagaoka et al., 2010; using *Bombyx mori*, a more specialized herbivore).

Calcium oxalate crystals in isolation may not be an effective deterrent to herbivores but instead may rely on a synergism with other defense traits, complicating investigations of the impacts of crystals on herbivores. In a 2014 paper, Konno and colleagues found that kiwifruit raphides and cysteine proteases, a class of defense metabolites, worked together at biologically relevant levels to decrease the growth and increase the mortality of eri silkmoth (*Samia ricini*) larvae to a degree that was greater than the sum of the impacts of the two defenses on their own, a phenomenon they dubbed the “needle effect.” Thus, the negligible effect of CaOx crystals on some herbivores may be because raphides are an effective defense only in the presence of secondary metabolism (Konno et al., 2014). The work by Konno also demonstrated that it was specifically the needle shape of the crystals that facilitated this synergism, as the larvae fed amorphous crystal grains and metabolites did not experience higher mortality than those fed just the metabolites.

Work by Volk and colleagues proposed that different crystal morphologies may play different functions in plants, and thus the aforementioned hypotheses may not be mutually exclusive (Volk et al., 2002). For instance, the works cited above found defensive properties of raphide needle crystals and prismatic crystals (Korth et al., 2006; Konno et al., 2014), whereas neither druses nor crystal sand morphotypes are found to decrease herbivore growth (Nagaoka et al., 2010). Based on this literature, we might expect that the formation of druse and crystal sand morphologies would be related to storage and would be induced by calcium levels in soil to act as a form of calcium storage, while raphide or prismatic morphologies additionally or alternatively provide defense for plants. Determining the degree to which CaOx is constitutively versus facultatively expressed via environmental induction, either by herbivory or by soil calcium, may help elucidate the functions of these widespread crystals, especially when paired with experimental manipulations of crystal presence in an herbivore's diet.

*Vitis riparia* Michx. (Vitaceae), or riverbank grape, presents an opportune system in which to test the ecology and inducibility of CaOx crystals because their organs contain at least two distinct crystal morphologies: raphide bundles and druses (Webb et al., 1995; Jáuregui‐Zúñiga et al., 2003; Figure 1A). Raphides and druses can be found in predictable localities within grape leaves; raphides are sporadically distributed throughout the mesophyll tissue in idioblasts, while druses are exclusively found in bundle sheath cells clustered in rows along veins in grape leaves (Webb et al., 1995; Arnott and Webb, 2000; Kolyva et al., 2023; Figure 1A). This differential locality may hint at differential roles, which suggests that raphides are distributed throughout the leaf to maximize their defensive capacity, whereas druses form near vascular tissue where calcium is deposited. Previous studies of calcium oxalate crystals have largely failed to test for the existence of alternate roles for different crystal morphologies.

**Figure 1:**
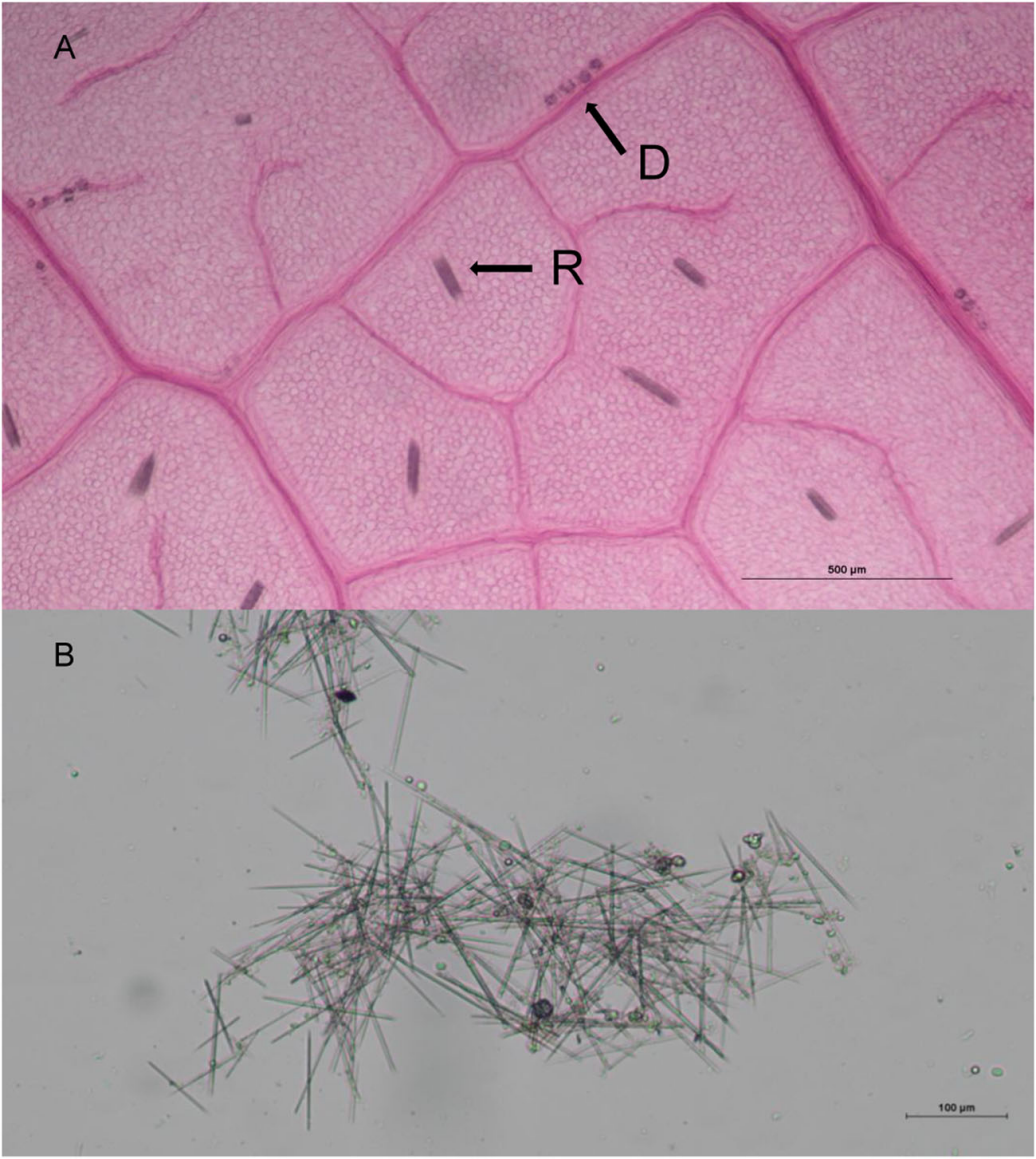
*Vitis riparia* calcium oxalate crystals (A) within leaf tissue stained with 0.5% Safranin O dye (bar = 500 µm) and (B) isolated from ground leaf tissue (bar = 100 µm). Structures labeled D are druse crystals, while those labeled R are raphide bundles.

Here, we provide experimental tests of the inducibility and function of crystal types in riverbank grape. First, we explored the causes of crystal formation, testing whether environmental stimuli influence their formation. We performed a greenhouse‐based experimental manipulation to study (1) whether different calcium oxalate crystal morphologies (raphides and druses) are differentially inducible through calcium soil addition or simulated herbivory in *V. riparia*. If calcium oxalate crystals are inducible in *V. riparia*, we predicted that raphides are formed in response to herbivory, whereas druses are induced through calcium addition. Second, we conducted a series of bioassays using generalist herbivores with a tightly controlled factorial design to test whether crystals provide antiherbivore defense. We specifically asked whether (2.1) herbivorous arthropod growth is impacted by the presence of *V. riparia* raphides in their diet, (2.2) crystal shape determines the impact of CaOx on herbivore growth, and (2.3) raphides operate synergistically with plant metabolites to the detriment of herbivores. We predicted that *V. riparia*‐derived CaOx crystals will be a more effective defense in the presence of chemical defenses than they are on their own. In comparison to previous work on the trophic ecology of CaOx crystals, this bioassay strategy enabled us to examine the long‐term impacts of different crystal morphologies on herbivores in order to more closely replicate the realities of sustained crystal diets in the wild.

## MATERIALS AND METHODS

### Inducibility of calcium oxalate crystals in *Vitis riparia*

We rooted genetically identical *V. riparia* cuttings collected in Michigan, USA, in a common cold‐room environment, with heating pads under the pots to stimulate root growth and no light, in the spring of 2024. Once the vines had begun to grow roots and buds, we moved them to a shared greenhouse bench with ambient light conditions (early summer in central Michigan). We then separated the cuttings into three experimental treatments: calcium addition, simulated herbivory, and control. Calcium addition plants were watered with a solution of 15 mM calcium chloride twice a week, achieving a soil calcium concentration of ~1700 ppm (vs. ~100 ppm in the control treatment; methods and concentrations adapted from Volk et al., 2002). Herbivory treatment plants received the same amount of water without calcium but were sprayed with 100 mg jasmonic acid dissolved in 250 mL 0.1% acetone twice during the 6 wk growing period. We also simulated herbivory on plants in the herbivory treatment by removing one‐third of the area of each mature leaf twice in the growing period, at the same time points as the hormone treatment (methods adapted from van Kleunen et al., 2004). All plants from all treatments were handled when the simulated herbivory treatment was applied. Control treatment plants received the same amount of water and were sprayed with a control spray made up of 0.1% acetone in water.

After the 6 wk growing period, we removed one mature, undamaged leaf from each vine (approximately the first fully expanded leaf from the growing end of the vine) and dried it in a drying oven at 60°C until fully dehydrated. We cleared, stained, and mounted leaves using a procedure adapted from Machesky et al. (2025). In brief, we soaked the leaves in sodium hydroxide until they became transparent, washed them in distilled water, and soaked them in bleach until decolorized. We then dehydrated leaves in increasing concentrations of ethanol, up to pure ethanol, and soaked them in 0.5% Safranin O dye in ethanol to stain the leaves. After washing off excess dye in ethanol, we fixed the leaves in cedar oil and examined them under a compound microscope at 4× magnification. We took pictures of the samples with the microscope and then counted the number of raphide bundles and druses visible within the resulting standardized image size.

#### Statistical analyses

To test whether calcium oxalate crystals are inducible, we constructed generalized linear models in which the predictor is treatment (control, calcium addition, or simulated herbivory) and response variables are either raphide or druse counts (per a standardized area), with observer as a random effect. Because the data, especially in the case of the druse counts, were overdispersed and zero‐inflated, we implemented models with a negative binomial correlation structure with a zero‐inflation extension using the R function “glmmTMB” in the package of the same name (Brooks et al., 2017).

### Effects of *Vitis riparia* leaf calcium oxalate crystals on herbivores

To test the defensive function of *V. riparia* CaOx crystals, we performed two bioassay experiments.

#### Bioassay 1 design

In the first bioassay, we fed leaves sourced from plants subjected to the control and calcium induction treatments described above to generalist herbivore larvae of *Spodoptera exigua* (Frontier Scientific Services Agriculture, Newark, Delaware, USA). Each larva was enclosed in a cup with a single *V. riparia* leaf for 4 d and stored in an incubator, which was set to 21°C with 9 h of light. We weighed the larvae each day of the bioassay to measure biomass gain/loss.

#### Bioassay 2 design

The second bioassay was a factorial no‐choice bioassay varying the presence of riverbank grape leaf CaOx crystals, amorphous CaOx crystals, and leaf chemistry in artificial diet. We used the same herbivore, *S. exigua*, in this experiment.

##### Acquiring chemical and crystal diet additives

To control for the impact of other defense traits present in *V. riparia* leaves on herbivores, crystals and leaf metabolites were extracted from wild riverbank grape leaf material and introduced into the artificial diet. To obtain riverbank grape leaf metabolite extracts for the bioassay's chemistry treatment, we developed a metabolite extraction procedure using standard methods in the field (e.g., Sedio et al., 2021) and our previously published knowledge of classes of compounds hypothesized to contribute to defense in wild grape, such as flavonols, anthocyanins, and other phenolic compounds, as well as terpenes (Fernandes et al., 2013; Kedrina‐Okutan et al., 2018; Pintać et al., 2019; Graham et al., 2023). We collected mature, undamaged leaves from wild *V. riparia* vines near Ann Arbor, Michigan, in the summer of 2023 and dried them at room temperature using silica gel beads. Once the leaves were fully dry, we ground them into a fine powder using a mortar and pestle. We then submerged the powdered leaf material in 100% methanol for 10 min to extract leaf metabolites and then centrifuged these extracts so that solid leaf material could be separated from methanol supernatant. We stored the supernatant extract at −20°C until just prior to the bioassay. In the days leading up to the bioassay, we once again centrifuged the extract to remove any remaining solid particles. We then let the methanol evaporate off in a fume hood at room temperature for ~48 h, leaving behind a leaf metabolite residue. Finally, we resuspended this residue in 1% sunflower seed oil in DI water, with the goal of including both polar and nonpolar compounds in this chemical stock solution. This resulted in a leaf metabolite extract of ~47.44 g/L (w/v).

To create our *V. riparia*‐derived calcium oxalate crystal treatment, we isolated CaOx crystals from the leaf pulp pellet that resulted from the chemical extraction and centrifugation of ground leaf material described above. To extract the crystals from this pellet, we used a modified version of the CaOx extraction procedure described in Konno et al. (2014). In brief, we separated unwanted leaf material from calcium oxalate by using a solution of 6.35 M cesium chloride and 0.4 M calcium chloride, with a density of ~1.8 g/mL. This solution had a density intermediate between the densities of the unwanted leaf material and the CaOx crystals and, when centrifuged, produced a pellet of CaOx crystals and a surface layer of leaf pulp. We repeated this dilution and centrifugation procedure three times, each time discarding the unwanted leaf material layer. We then washed the pellet an additional three times with distilled water to obtain uncontaminated calcium oxalate crystals. We then allowed the pellet to dry so that we could accurately weigh the crystals into the artificial diet. Examination of extracted crystals under light microscopy indicated that the initial drying and grinding of the riverbank grape leaf material did not damage the crystals. However, the raphide crystals were loosened from their bundles (Figure 1B). The vast majority of crystals extracted in this manner were raphide needles, with some druses dispersed throughout. Amorphous CaOx crystals were purchased as a purified reagent (Thermo Scientific Chemicals, Pittsburgh, Pennsylvania, USA). These crystals are most similar in form to sand crystals and do not exhibit a needle shape.

##### Experimental setup

**Figure 2:**
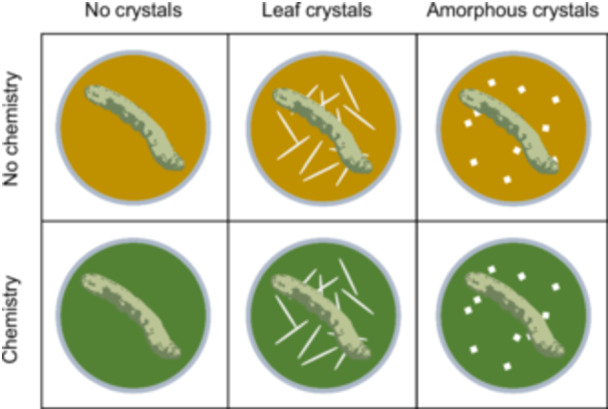
Factorial design of the second bioassay, allowing us to test the impacts of crystal shape and presence and of grape chemical presence on *Spodoptera exigua* growth.

To test the individual and combined effects of calcium oxalate crystals and leaf chemistry on herbivore fitness, we developed our own bioassay methodology in which herbivores were enclosed in individual plastic cups containing one of six possible artificial diet treatments (Figure 2). Each diet cup contained 5 mL of general lepidopteran artificial diet (Frontier Scientific Services Agriculture) prepared in one large batch according to the instructions provided and dispensed into the cup. While the diet in each cup was still liquid, we thoroughly mixed in 1 mL of treatment stock solution. We then allowed the diet to set and stored the cups at 4°C until the following day, when we set up the bioassay.

The treatment stock solutions were designed so that dispensing 1 mL of solution into the diet cups would introduce 2.85 mg of CaOx crystals (either *V. riparia*‐derived or amorphous) and/or 47.44 mg of leaf metabolites. All treatment solutions had a base of 1% sunflower seed oil in DI water. After allowing the diet cups to warm to room temperature, we introduced a single third (approximately) instar *S. exigua* larva (9–15 mg in mass, *x̄* = 11.7 mg) to each diet cup. We allowed them to feed until pupation, which took 11–21 d depending on the treatment. The cups were stored within trays (each holding 30 cups) in the incubator, which was again set to 21°C with 9 h of light. Treatment cups were randomized within trays at the outset of the experiment, but the trays were consistently kept in the same incubator locations. We included more than enough diet for the caterpillars to get to pupation; no caterpillars ate all their provided diet.

Every day during the bioassay, we weighed each larva and surveyed them for survival and/or pupation. Pupae were weighed in the same order each day to maintain roughly consistent feeding intervals across days. We collected all pupae and weighed them to get a final wet mass measurement.

##### Statistical analyses

In both bioassay analyses, we aimed to capture a holistic picture of the impacts of our treatments on the herbivores by analyzing multiple metrics of herbivore success. In bioassay 1, we looked at the percent change in mass of the herbivores over the 4 d bioassay period, as well as larval survival (a binary variable, 0/1), between the two treatment groups (control and calcium addition). In bioassay 2, we looked at the maximum mass the herbivores achieved, the number of days it took for them to reach this maximum mass, whether they survived to pupation, and the mass of the pupae if they did pupate. We converted the raw larval mass data to percent growth to account for any differences in the initial masses of the larvae:

$$\mathrm{percent}\;\mathrm{growth}=\frac{m_{\text{final}}-m_{\text{initial}}}{m_{\text{initial}}}\;\times 100$$

We also excluded larvae from the analyses that perished without any evidence of having eaten the artificial diet (14 larvae across all treatments in bioassay 2).

We used analysis of variance to compare maximum larval and pupal masses between treatments, and we used binomial logistic regressions to assess survival. For bioassay 2, all models included terms for individual effects of CaOx treatment (leaf‐derived crystals or amorphous crystals) and chemistry, as well as CaOx‐chemistry interaction terms. Additionally, in preliminary analyses we included tray as a random effect in mixed‐effects models using the package lme4, but discovered no qualitative impact on the results, so we excluded this random effect from the final models. All analyses were performed in R version 4.2.1 (R Core Team, 2021).

## RESULTS

### Are different calcium oxalate crystal morphologies differentially inducible through calcium soil addition or simulated herbivory in *V. riparia*?

**Figure 3:**
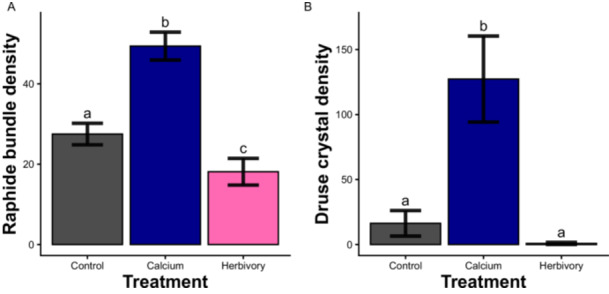
Average leaf (A) raphide bundle and (B) druse crystal densities from vines subjected to the three induction treatments. Error bars represent standard error in measurements calculated for the raw density data. Bars labeled with different letters have model estimates significantly different from each other at the *p* = 0.05 level. Note that the *y*‐axes between panels A and B are markedly different, indicating the high density differences between raphide and druse crytals in response to our treatments.

The calcium addition treatment had a significant positive impact on the density of both raphide bundles and druses in *V. riparia* leaves (*p* <<< 0.05 for both; Figure 3). Contrary to our expectations, vines in the herbivory treatment had a lower density of raphide bundles than those in the control treatment (*p* = 0.001). There was no effect of simulated herbivory on druses, and very few leaves from the control or herbivory treatments even had druses.

### Do *Vitis riparia*‐derived calcium oxalate crystals impede herbivore growth?

**Figure 4:**
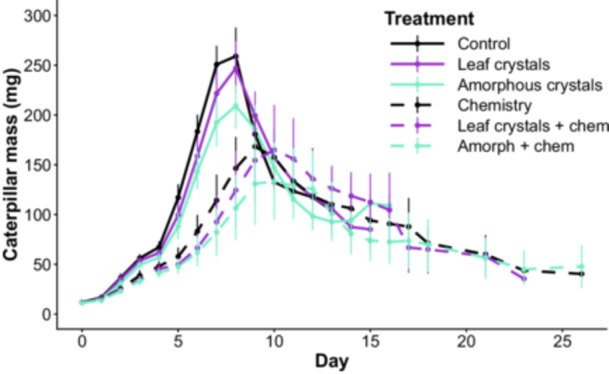
Average growth curves of larvae by treatment. Error bars represent confidence limits for the caterpillar mass means per treatment at each time point.

**Figure 5:**
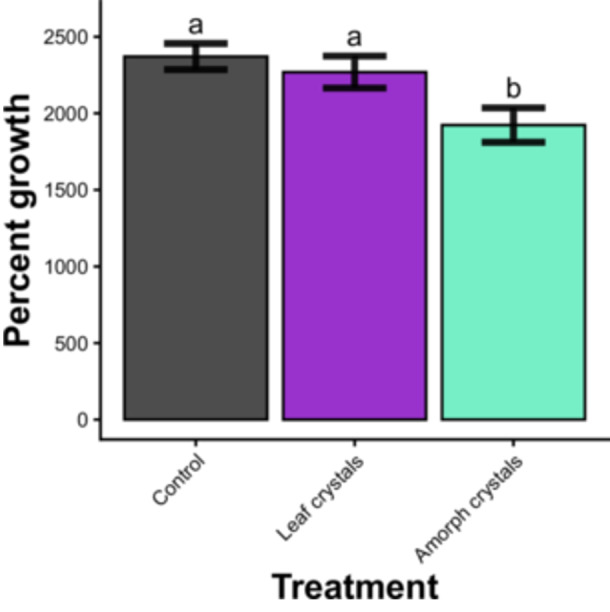
Effect of crystal shape on percent growth of caterpillars. Error bars represent standard error in measurements calculated for percent growth data. Bars labeled with different letters have model estimates significantly different from each other at the *p* = 0.05 level.

In bioassay 1, we found no effect of the calcium treatment on percent mass gain (*p* = 0.829; Appendix S1) or survival (*p* = 0.094) of larvae fed leaves from the control and calcium treatments in the induction experiment. In bioassay 2, we similarly found no significant impact of *V. riparia* leaf‐derived CaOx crystals on any of our metrics of herbivore success. Larvae in this treatment reached a similar average maximum percent growth (control = 2371% ± 85% growth; *V. riparia* CaOx = 2269% ± 104% growth; Figure 5) and pupal mass (control = 123 ± 5 mg, *V. riparia* CaOx = 110 ± 6 mg) as the control treatment and were not significantly more likely to pupate (Table 1). Because the results for the maximum percent growth and wet pupal mass were qualitatively identical, we have elected to graph only the percent growth results. Additionally, the larvae fed *V. riparia* leaf‐derived crystals reached their maximum mass at the same time as the control larvae, meaning that the calcium oxalate crystals did not slow their growth (Figure 4).

**Table 1:** Effects of treatment on the odds of larval survival to pupation. Note that the total and deceased larvae counts do not include the five larvae per treatment that were sacrificed. Significance comparisons are made to the control in the case of the additive effects, while the interaction effects are treated as interactions in the models.

Treatment	Total larvae in treatment (*n*)	Count of larvae that died	Log odds of survival	SE	*p*
Control	22	3	0.463	0.621	–
*Vitis riparia* leaf crystals	22	5	1.22	0.803	0.438
Leaf chemistry	24	14	−0.595	0.747	0.00347**
Amorphous crystals	22	14	−0.560	0.763	0.00162**
*V. riparia* leaf crystals + chemistry	15	14	−1.872	0.981	0.439
Amorphous crystals + chemistry	21	15	−0.9163	0.787	0.0661

### Does crystal shape determine the impact of calcium oxalate crystals on herbivore growth?

In bioassay 2, contrary to our prediction, we found that amorphous CaOx crystals had a significant negative impact on herbivore growth in comparison to the control treatment. This was true for both the average maximum percent growth of the larvae during the bioassay (*p* = 0.01; Figure 5) and the average pupal masses (*p* = 0.007).

### Do CaOx crystals synergize with *Vitis riparia*‐derived metabolites to the detriment of herbivores?

**Figure 6:**
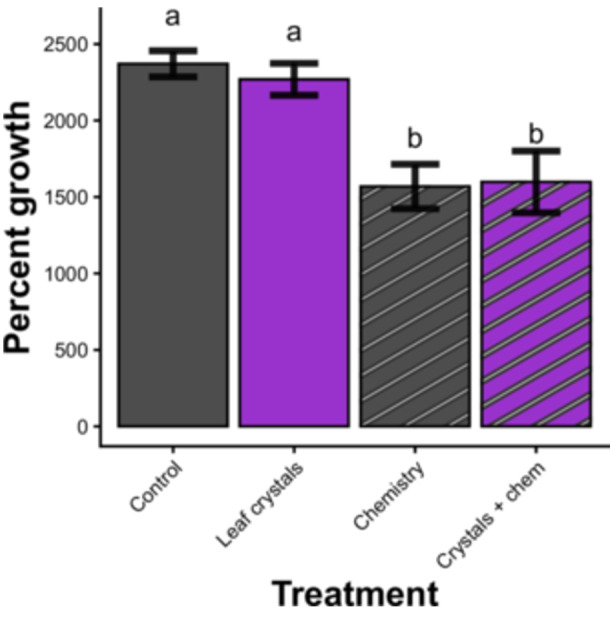
Lack of a synergistic effect of *Vitis riparia* leaf‐derived calcium oxalate crystals and grape leaf chemistry on caterpillar percent growth. Error bars represent standard error in measurements calculated for percent growth data. Bars labeled with different letters have model estimates significantly different from each other at the *p* = 0.05 level.

We did not detect a significant interaction between *V. riparia* leaf‐derived CaOx crystals and leaf chemistry in relation to herbivore survival or our other metrics of herbivore success. Leaf chemistry did decrease the maximum percent growth the larvae achieved in comparison to the control (*p* < 0.00001; Figure 6), and larvae in the leaf chemistry treatment had lower odds of surviving to pupation (*p* = 0.003; Table 1). Also, among the few larvae that made it to pupation under the chemistry treatment, those pupae were significantly smaller than the control pupae (*p* < 0.0001). We found no evidence for a synergism between chemistry and amorphous CaOx crystals.

Larvae in the chemistry treatment also reached their maximum mass more slowly than those in the control treatment (*p* = 0.002). It took an average of ~9 d for the control, leaf crytsal, and amorphous treatment larvae to reach their maximum mass, whereas larvae in the chemistry treatment took an average of ~2 d longer (Figure 4).

## DISCUSSION

The causes and consequences of calcium oxalate crystal presence in plants have sparked significant debate, which has recently been renewed by several review articles (Paiva, 2019, 2021). Here, we demonstrate that CaOx crystal density is inducible via calcium soil addition in *V. riparia*, but not through simulated herbivory. Additionally, we show that *V. riparia* calcium oxalate crystals do not impact the feeding of a generalist herbivore, neither in situ nor ex situ, and do not seem to enhance the defensive function of *V. riparia* secondary chemistry. The results of this study challenge the assumption that raphide calcium oxalate crystals function as a growth reducer for chewing herbivores across plant species, and fail to support the hypothesized “needle effect” synergism of needle‐shaped crystals and chemical defense (Konno et al., 2014).

This work joins the growing body of research suggesting that foliar calcium oxalate crystals are overemphasized as a defense against insect herbivores (Paiva, 2021). Why do *V. riparia* crystals, which primarily take the form of bundles of sharp raphides, fail to impact the growth of this generalist herbivore? One likely culprit is the peritrophic matrix, a semipermeable chitin‐based barrier in the guts of many chewing insects that protects their soft interiors from abrasion caused by ingested materials and microorganisms (Terra, 2001; Mohan et al., 2006; Paiva, 2021). Indeed, Paiva (2021) argues that the presence of the peritrophic matrix prevents CaOx from harming the midguts of *Spodoptera exigua*, as evidenced by the lack of an effect of CaOx on the peritrophic membranes of larvae of that species reported in Park et al. (2009). Although this mechanism of tolerance to ingested CaOx crystals has been proposed, to our knowledge it has yet to be directly tested by comparing insect herbivores with and without intact peritrophic matrices. Additionally, it is possible that the herbivores in our second bioassay experiment were able to avoid the crystals. Some evidence suggests that mature caterpillars are able to sense CaOx crystals and avoid them (Doege, 2003), an advantage that small arthropod herbivores might have over mammal herbivores. We hypothesize that the caterpillars in our study were able to avoid the larger raphide needles and druses, but not the smaller amorphous crystals used for the crystal shape control, consistent with the result that amorphous crystals had a negative impact on herbivore growth. Since no herbivores ate all the provided leaf (bioassay 1) or diet (bioassay 2), it is possible that the larvae were not forced by food scarcity to eat the crystals. In the first 5 d of eating, the growth curves of caterpillars on the control, raphide, and amorphous crystal treatments closely followed each other, implying that the impact of the amorphous CaOx in the diet was not immediate. Gradually, the amorphous crystal growth curve began to separate from the other two treatment curves, which may indicate that the impact of the crystals is cumulative and/or the result of learning that the crystals are unpleasant to ingest. It is important to note that while mandible wear may be a component of this gradual effect, *S. exigua* caterpillars shed their mandibles upon transition between instars, meaning that any growth impact of worn mandibles on the larvae is likely only temporary.

In addition, *V. riparia*‐derived CaOx crystals did not modify the effect of leaf chemistry on herbivores in bioassay 2. This is in contrast to the results of Konno et al. (2014), where kiwi‐derived raphides had a negative impact on silkmoth caterpillar growth within a 24 h period, though they found no impact on caterpillar mortality. There are several reasons why our results may contrast with those of Konno et al. (2014). One key factor is that the caterpillars used in the 2014 study were neonates whereas our larvae were around third instar when placed on the diet. It is thus possible that raphides are a detriment only to young lepidopteran larvae and that at larger sizes the larvae can tolerate the presence of crystals. Additionally, the raphides in the Konno bioassay were applied to the surface of castor oil leaves, rather than mixed into the artificial diet, leaving the possibility that the crystals produced a physical pre‐ingestive effect on the neonate larvae that was absent when the raphides were mixed into our diet cups. If the peritrophic matrix shielded the internal structures of the larvae in our study, and those larvae did not come into contact with the needle‐crystals externally, that could explain the discrepancy between our results and the apparent needle effect found by Konno and colleagues. Finally, the chemical compound fed to larvae in conjunction with CaOx crystals by Konno et al. (2014) was an isolated cysteine protease called bromelain, whereas we extracted compounds, regardless of chemical identity, from grape leaf tissue to simulate the cocktail of compounds that herbivores encounter when they consume leaf tissue in the wild. Ultimately, our results do not support a pre‐ or post‐digestion defense function for calcium oxalate crystals in riverbank grape.

In general, we saw that the presence and abundance of druse crystals in *V. riparia* leaves across all treatments of the induction experiment were more variable than those of raphides, suggesting that druses are more plastic and perhaps more ephemeral than raphide bundles. We predicted that druse crystals would be sensitive to calcium levels in the soil and that raphides would not be; our finding that the densities of both crystal types increase in calcium‐rich soil supports the former but not the latter prediction. Calcium oxalate crystals have long been hypothesized to be a dynamic storage system for calcium in plants (Volk et al., 2002; Franceschi and Nakata, 2005; Paiva, 2019). Mounting evidence suggests that there are both environmental and genetic components to the phenotypic expression of CaOx crystals (Webb, 1991). Calcium oxalate crystal formation can be genetically engineered in plant species that do not naturally form these crystals (Nakata, 2012), and evidence suggests that different crystal types are formed via different biosynthetic pathways (Nakata and McConn, 2007). In the case of raphides—bundles of needle‐crystals that are formed in specialized vacuoles called idioblasts—cell direction is almost definitely implicated (Franceschi and Nakata, 2005). However, our understanding of the genetic regulation of crystal morphology and abundance is still nascent. Another investigation of differential inducibility of CaOx crystal morphologies found that moving *Pistia stratiotes* plants to calcium‐scarce conditions from control concentrations caused a decrease in druses but no change in raphides, demonstrating that druses seem to be more responsive to soil calcium than raphide bundles in that system (Volk et al., 2002). In that study, both types of crystals were more prevalent and larger in high‐calcium conditions. Similarly to those of *V. riparia*, druse crystals of *P. stratiotes* form near veins, whereas raphides are in the aerenchyma (Volk et al., 2002). The slight decrease in the presence of CaOx crystals of both types in response to the simulated herbivory treatment (though not significant in the case of the druse morphology) may be a result of decreased transpiration caused by the loss of leaf area, decreasing the transport of calcium in the xylem from the roots to the shoots of the vines (White and Broadley, 2003). We note that the leaves that were sampled for crystal quantification were specifically undamaged, but that does not rule out the possibility of systemic impacts of leaf area removal on transpiration rates.

Although our experimental evidence supports the hypothesis that CaOx has a dynamic storage function in plants, it is worth noting that a small but growing body of work has implicated calcium oxalate crystals in “alarm photosynthesis,” whereby carbon is collected and stored as CaOx crystals (Tooulakou et al., 2016a, 2016b). In drought conditions when stomata are closed, the crystals are hypothesized to be degraded by oxalate oxidase to provide CO_2_ for photosynthesis, with H_2_O_2_ and Ca^2+^ ions as byproducts (Tooulakou et al., 2016a). In studies of pigweed, levels of CaOx crystals fluctuated throughout the day and were shown to decrease when plants encountered artificial CO_2_ stress conditions (Tooulakou et al., 2016a, 2019). The location of *V. riparia* druses in bundle sheath cells near leaf veins supports their role in this biosynthetic pathway. Indeed, work on *V. vinifera*, a cultivated congener of *V. riparia*, found that druse crystal, but not raphide crystal, densities decreased slightly during the day, and more dramatically after application of a water stress treatment (Kolyva et al., 2023). Water availability was not variable across our treatments, and we expect that variation in crystal abundance in our plants was minimally influenced by water availability, and instead that we primarily measured variation due to calcium availability and leaf damage.

## CONCLUSIONS

Our results add to the growing body of scholarship elucidating the causes and consequences of calcium oxalate crystal formation in plants, explicitly testing the inducibility and putative defensive function of these mysterious structures. Our study provides evidence that calcium oxalate production can be induced by excess calcium and that *V. riparia*‐derived crystals do not impair the growth and survival of a generalist chewing herbivore. Across plants, the variety of environmental conditions that produce calcium oxalate crystals, as well as their failure to provide consistent antiherbivore defense across plant and herbivore species, suggests that different crystal morphologies have different functions. This generates new questions about the context dependency of calcium oxalate ecology.

## AUTHOR CONTRIBUTIONS

C.D.K.G., S.M., and M.G.W. conceptualized and designed the study. C.D.K.G., S.M., and A.L.Y. ran the experiments and collected all data. C.D.K.G. analyzed the data and wrote the manuscript. All authors discussed the results and implications of the work and revised the manuscript.

## Supporting information


**Appendix S1.** Results for bioassay 1.

## Data Availability

All data used in this study are publicly available on Dryad at https://doi.org/10.5061/dryad.d7wm37qfb.
