# Supplementary material for: Characterizing the causes and consequences of calcium oxalate crystal presence in Vitis riparia
Source: Am J Bot. 2026 Mar 9;113(3):e70173. doi: 10.1002/ajb2.70173 (PMC13003718; doi:10.1002/ajb2.70173)
Supplement: Supplementary file 1 — Appendix S1. Results for bioassay 1. [file AJB2-113-e70173-s001.docx]

Graham et al.—American Journal of Botany 2026 – Appendix S1


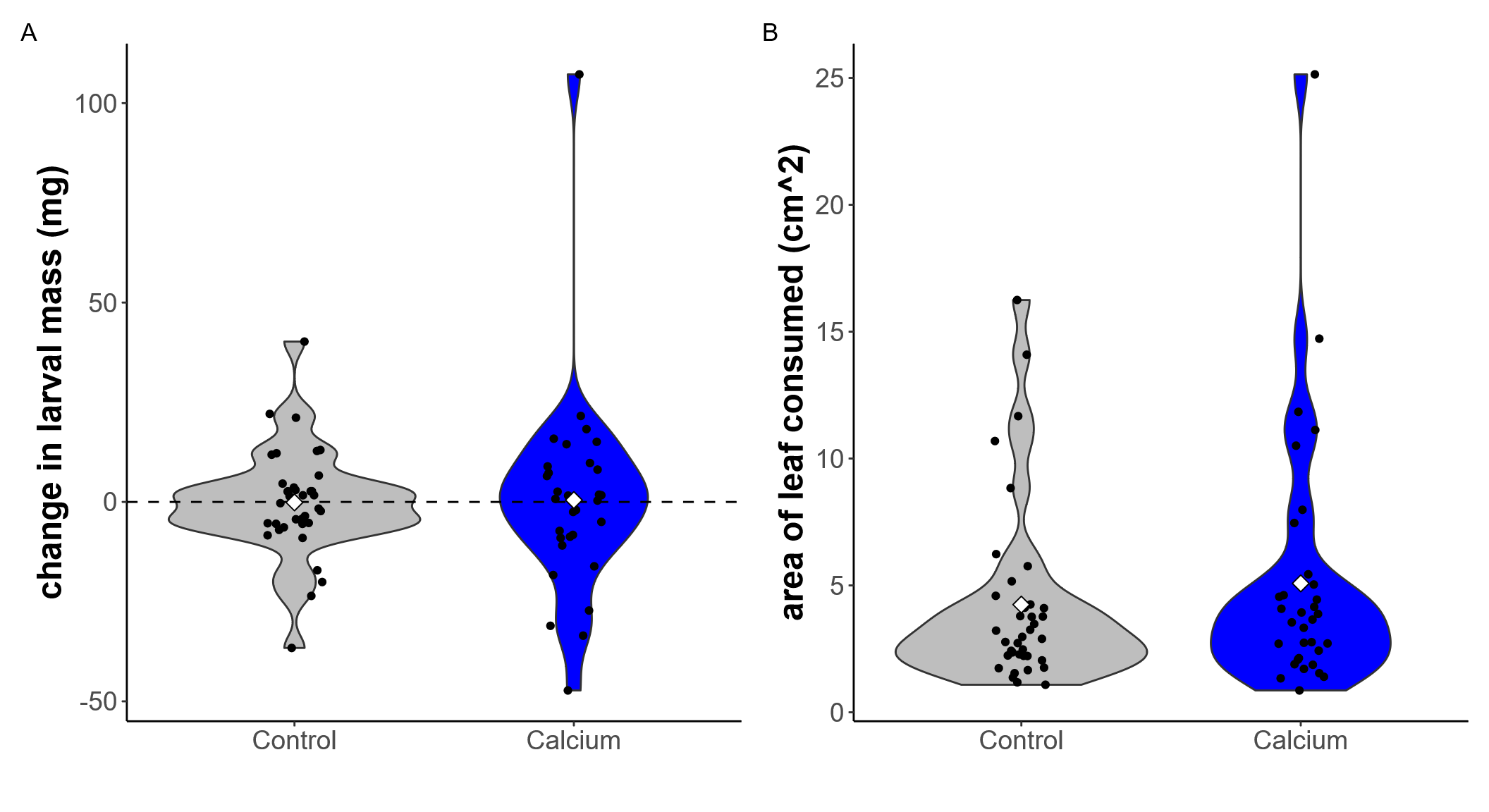


**Figure S1**. Results for bioassay 1, demonstrating no difference in change in mass over four days for larvae fed on control and calcium treatment leaves. Treatment group means are plotted as white diamonds. Each black point is the raw value for an individual larva.
